# Artificial intelligence demonstrates comparable diagnostic accuracy to radiologists for anterior cruciate ligament tears on MRI: A systematic review and meta‐analysis

**DOI:** 10.1002/ksa.70478

**Published:** 2026-06-30

**Authors:** Gabriel Moraes de Oliveira, Serafina Zotter, Brian Tao, Ana Beatriz Nardelli da Silva, Maria Mascarenhas, Chilan B. G. Leite, Guilherme Almeida, Nacime Mansur, Giovanna Medina

**Affiliations:** ^1^ Paulista School of Medicine Federal University of Sao Paulo (EPM‐UNIFESP) São Paulo Brazil; ^2^ Massachusetts General Hospital Harvard Medical School Boston USA; ^3^ Boston University Chobanian & Averdisian School of Medicine Boston Massachusetts USA; ^4^ University of Bradford Bradford UK; ^5^ Santa Casa de São Paulo School of Medical Sciences São Paulo Brazil; ^6^ Brigham and Women's Hospital, Harvard Medical School Boston Massachusetts USA; ^7^ Federal University of Sergipe Aracaju Brazil; ^8^ Medstar Union Memorial Hospital Baltimore Maryland USA

**Keywords:** ACL, artificial intelligence, deep learning, machine learning, neural networks, primary care

## Abstract

**Purpose:**

Anterior cruciate ligament (ACL) tears are among the most common knee injuries, accounting for half of all knee ligament injuries. Magnetic resonance imaging (MRI) is the standard for diagnosing ACL tears, but its interpretation is experience‐dependent. Artificial intelligence (AI), particularly deep learning (DL), has emerged as a transformative tool in medical imaging, offering advanced pattern recognition for detecting abnormalities.

**Methods:**

This study systematically reviews and meta‐analyses the diagnostic accuracy of AI in detecting ACL tears, comparing with radiologists' performance. Following Preferred Reporting Items for Systematic Reviews and Meta‐Analyses of Diagnostic Test Accuracy Studies (PRISMA‐DTA) guidelines, a comprehensive search of Embase, PubMed and Cochrane databases identified 247 articles, with 40 studies included in qualitative synthesis and 31 in meta‐analysis, encompassing 1192 cases.

**Results:**

In the overall pooled analysis, AI demonstrated a sensitivity of 0.94 (95% confidence interval [CI]: 0.93–0.95) and specificity of 0.93 (95% CI: 0.92–0.93). In the subgroup comparing AI directly to radiologists, AI demonstrated pooled sensitivity and specificity of 0.90 (95% CI: 0.88–0.92) and 0.91 (95% CI: 0.89–0.92), respectively, while radiologists showed 0.85 (95% CI: 0.83–0.87) and 0.90 (95% CI: 0.88–0.91). Heterogeneity varied, with moderate to high heterogeneity in the analyses. Summary receiver operating characteristic (sROC) analysis indicated no significant difference between AI and radiologists in diagnostic accuracy (*p* = 0.782).

**Conclusion:**

These findings suggest that AI can match human diagnostic performance for ACL tears, offering advantages such as reduced costs, faster results and decreased physician burden. Despite variability in study settings, AI shows promise as a reliable diagnostic tool in knee imaging.

**Level of Evidence:**

Level II.

**Abbreviations**
ACL: anterior cruciate ligament
AI: artificial intelligence
AUC: area under the curve
CNNs: convolutional neural networks
DL: deep learning
FN: false negatives
FP: false positives
MCC: Matthew's correlation coefficient
MRI: magnetic resonance imaging
NPV: negative predictive value
PCPs: primary care providers
PPV: positive predictive value
PRISMA‐DTA: Preferred Reporting Items for Systematic Reviews and Meta‐Analyses of Diagnostic Test Accuracy Studies
sROC: summary receiver operating characteristic
TN: true negatives
TP: true positives
XAI: explainable AI

## INTRODUCTION

Anterior cruciate ligament (ACL) tear is one of the most common knee injuries, accounting for almost half of all knee ligament injuries and affecting over 200,000 individuals annually in the United States [28, 42]. Early and accurate diagnosis of ACL tears is paramount as timely intervention can prevent further joint damage, such as chondral or meniscal injuries. A delayed or overlooked diagnosis often results in increased time from injury to surgery and is positively correlated with an increased risk of developing osteoarthritis later in life [12]. Magnetic resonance imaging (MRI) is the standard method for evaluating knee injuries, offering high sensitivity and specificity in detecting ACL injuries [3, 49]. However, this image‐based diagnosis of ACL injuries is performed by visual assessment of shape and signal characteristics, making accurate interpretation of knee MRI highly dependent on the experience of the reader [30]. This complexity underscores the necessity for more sophisticated diagnostic tools that can assist in the accurate assessment of ACL injuries.

In this context, artificial intelligence (AI) technologies have shown promising capabilities in medical imaging interpretation [2]. Deep learning (DL), a subset of AI focused on leveraging complex neural networks, is adept at recognizing intricate patterns in imaging data. Convolutional neural networks (CNNs), a class of deep neural networks, are especially effective in pixel classification tasks, making them ideal for medical image analysis [47, 60]. The application of AI in medical imaging, which has seen rapid expansion in recent years, includes a broad range of diagnostic tools that significantly enhance image analysis [20].

The development of AI‐driven diagnostic tools can substantially streamline the diagnostic process, reduce the likelihood of misdiagnosis and enhance educational outcomes for medical staff. Moreover, AI has the potential to support primary care providers (PCPs) by providing them with accurate and timely diagnostic assistance, enabling more informed decision‐making and democratizing high‐quality diagnostic capabilities, which could particularly benefit resource‐constrained settings, where access to specialized medical expertise may be limited.

AI's integration into medical practice is supported by a growing body of research that indicates AI can match and even surpass the diagnostic performance of human experts in certain scenarios [44]. Specifically within musculoskeletal imaging, prior work has demonstrated the successful development of DL models to detect various internal joint derangements on MRI, such as meniscus tears, cartilage defects and rotator cuff disorders [17, 63]. If AI can reliably identify ACL tears on MRI, it would represent a significant advancement in the field, reducing dependency on human interpretation and potentially speeding up the diagnostic process.

This systematic review and meta‐analysis aim to review and analyse the diagnostic accuracy of DL applications in the detection of ACL tears, exploring the overall performance of AI compared to traditional radiological assessments.

## METHODS

### Registration

This systematic review and meta‐analysis protocol was registered in the International Prospective Register of Systematic Reviews (CRD42025635943).

### Framework

The review was conducted and reported in accordance with the *Preferred Reporting Items for Systematic Reviews and Meta‐Analyses of Diagnostic Test Accuracy Studies* (PRISMA‐DTA) guidelines [41]. The Cochrane Handbook for Systematic Reviews of Diagnostic Test Accuracy Studies informed the methodological framework. The *Equity, Diversity and Inclusion (EDI) Statement* and the *Patient and Public Involvement Statement* are provided in the Methods section.

### Search strategy and data sources

Relevant studies were identified through systematic searches of three electronic databases: PubMed, Embase and the Cochrane Library. Searches were performed on 18 November 2024, with no date restrictions and limited to articles published in English. The search combined the following terms: *artificial intelligence* OR *machine learning* OR *deep learning* OR *neural network* AND *ACL* OR *anterior cruciate ligament* AND *MRI* OR *magnetic resonance*. The complete search strategies for each database are presented in Supporting Information S1: Figure 1. Reference lists of all included articles and related reviews were screened to identify additional eligible studies.

### Study selection

All records were imported into Covidence (Veritas Health Innovation) for screening and data management. Two reviewers independently screened titles, abstracts and full‐text articles for eligibility. When inclusion could not be determined from the title and abstract, the full text was reviewed. Discrepancies were resolved through discussion with a third reviewer until consensus was reached.

The following inclusion criteria were used: (i) MRI images were used to evaluate ACL injuries; (ii) diagnosis of ACL injury was performed using an AI model evaluated against an acceptable ground‐truth reference standard (i.e., arthroscopic/surgical confirmation, expert radiologist/orthopaedic surgeon consensus or established public datasets based on clinical reports); (iii) Articles were published in English. Exclusion criteria for articles were as follows: (i) studies not related to ACL injury diagnosis using AI model; (ii) non‐original research articles such as protocols, reviews and meta‐analysis.

### Data extraction

The data extracted included bibliographic details such as title, authors, abstract, year and month of publication, journal information and DOI. The methodological parameters of each study were also collected, including the target outputs and the number of MRI images used to train, validate and test the models. Performance metrics such as accuracy, sensitivity, specificity, prevalence, precision, area under the curve (AUC), Matthew's correlation coefficient (MCC), positive predictive value (PPV), negative predictive value (NPV), F1‐Score and kappa value were carefully recorded.

For studies that included a control group, comparative data between AI and traditional diagnostic methods (radiologists or arthroscopy) were documented, and additional performance metrics such as control group accuracy, sensitivity, specificity. This extensive data collection facilitated a detailed and nuanced analysis of the diagnostic performance of AI systems in comparison to traditional approaches, highlighting AI's capabilities and challenges in the clinical diagnosis of ACL tears.

As part of our meta‐analysis, data on true positives (TP), false positives (FP), false negatives (FN) and true negatives (TN) were extracted from the studies included. This extraction was critical for assessing the overall effectiveness of AI in diagnosing ACL tears, providing a quantifiable measure of AI performance across different clinical settings. Information regarding the source of MRI data, whether from MRNet or direct patient scans, and the type of AI technology used was also included.

### Quality assessment

As there is no widely accepted assessment tool for evaluating the quality of diagnostic assistance provided by AI, we used the QUADAS‐2 tool—the most commonly performed instrument for evaluating the quality of diagnostic trials [58]. To address this, two independent reviewers in our study evaluated each included study across four domains: patient selection, index test, reference standard and flow/timing. For each domain, the risk of bias was classified as low, high or unclear and applicability concerns were also assessed. Any disagreements between reviewers were resolved through discussion or by involving a third reviewer.

### Statistical analysis

To evaluate the performance of AI in diagnosing ACL tear, we summarized the sensitivity, specificity, diagnostic odds ratio (DOR) and 95% confidence intervals (CIs) based on the extracted TP, TN, FP and FN data. The summary receiver operating characteristic (sROC) curve was plotted and the AUC was calculated. The sROC curve is a graphical representation of the diagnostic performance of a continuous variable across multiple studies. The larger the area under each curve is, the better the diagnosis.

Publication bias was analysed using a funnel plot, with statistical significance for bias set at *p* < 0.05. The heterogeneity of the included studies was tested using the Cochrane *Q* test, with *I* ^2^ > 50% or a *p* value < 0.05, indicating significant heterogeneity. Heterogeneity was classified as low (*I* ^2^ < 25%), moderate (*I* ^2^ = 25%–75%) or high (*I* ^2^ > 75%). The quality of the studies included was assessed using Review Manager 5.4 (Cochrane Collaboration), and all statistics and analyses were completed using R for MacOS version 4.3.3 Software with the MIDAS package installed.

### Equity, diversity and inclusion statement

This systematic review explores the diagnostic accuracy of tests for ACL injuries, a condition affecting athletes and non‐athletes alike, regardless of gender, ethnicity or socioeconomic background. Although demographic reporting was limited in many of the included studies, our search strategy encompassed research conducted across multiple continents, ensuring a broad representation of clinical and geographic contexts.

Our author team reflects a commitment to diversity in both professional and personal dimensions. The group includes researchers and clinicians from North and South America, representing the fields of medicine, orthopaedics, radiology, sports medicine and data science. Women make up half of the author team, and members span early‐, mid‐ and senior‐career stages, each contributing complementary expertise in evidence synthesis, medical imaging and clinical practice.

We conducted every stage of this review, study selection, data extraction and analysis, with the shared goal of minimizing bias and fostering inclusivity. Decisions were made collaboratively, independent of institutional affiliation, country of origin or publication language. We recognize that equity, diversity and inclusion are ongoing responsibilities in research practice. By promoting fair access to mentorship, authorship and scholarly contribution, we aim to advance not only the quality of scientific evidence but also the inclusiveness of the academic community that produces it.

## RESULTS

### Systematic review

The literature search retrieved 247 articles, from which 72 duplicates were identified and removed. In the screening process, 175 studies were evaluated, and 126 studies were manually removed by reading the abstracts. After reading the full texts of the remaining articles, 9 studies were excluded, leading to the inclusion of 40 studies for the systematic review; details of the articles are shown in Supporting Information S1: Figure 2, and the flow chart of study selection is shown in Figure 1. Out of the 40 studies included [1, 4, 6, 7, 8, 9, 10, 11, 15, 16, 19, 24, 25, 26, 27, 29, 32, 33, 34, 35, 36, 37, 38, 39, 40, 45, 46, 48, 50, 51, 52, 53, 54, 55, 56, 57, 61, 62, 63, 65], 31 met the criteria for inclusion in the meta‐analysis [4, 6, 7, 8, 9, 10, 11, 15, 16, 19, 24, 25, 27, 29, 33, 34, 35, 37, 38, 40, 45, 46, 51, 52, 53, 55, 57, 59, 61, 62, 65]. Of these, 4 studies focused on evaluating the performance of AI compared to that of radiologists in diagnosing ACL tears [4, 9, 19, 29]. The remaining 27 studies either evaluated AI models strictly without a parallel comparison arm involving human readers, or they included a radiologist comparison but reported data that could not be pooled for meta‐analysis due to incompatible or missing statistical metrics.

**Figure 1:**
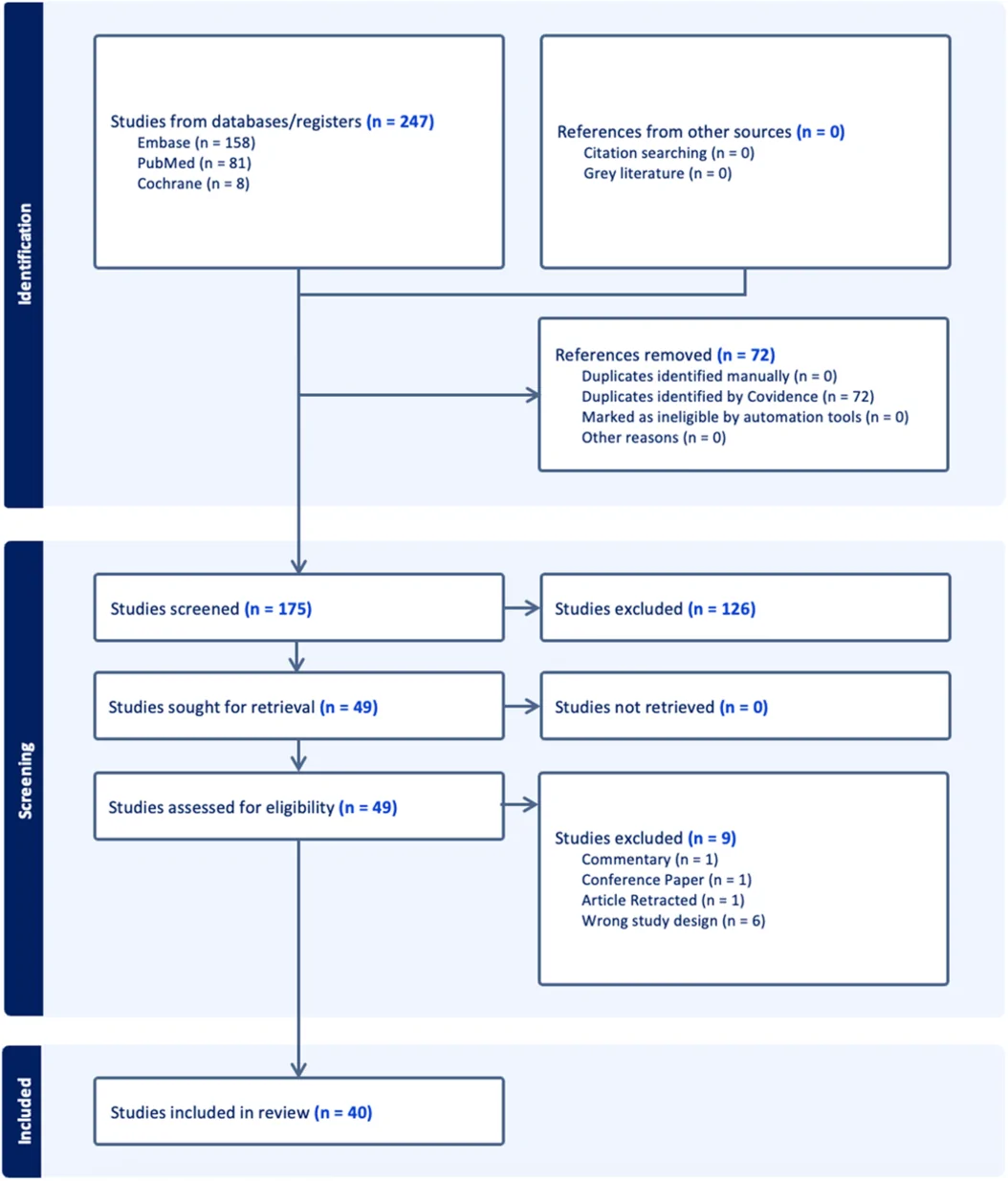
PRISMA flow diagram for study selection. PRISMA, Preferred Reporting Items for Systematic Reviews and Meta‐Analyses.

### Quality assessment

Of the 40 included studies, 34 had a low risk of bias in patient selection, while six studies raised concerns due to non‐randomized sampling or the use of MRI exams from multiple institutions, followed by surgery at a single institution. The absence of detailed information on whether sampling was consecutive or randomized may have introduced selection bias, limiting the generalizability of findings. Specifically, two studies were classified as high risk [7, 59] due to significant variability in patient recruitment methods, while four studies were categorized as unclear [19, 38, 46, 54] because of insufficient reporting on how participants were selected.

For the index test, bias was identified as high in one study. For the index test, most studies (36/40) were rated as low risk. Two studies were judged as unclear [4, 7] since it was not explicitly stated whether model predictions were assessed independently from the reference standard. Two studies [46, 59] were judged as having some concerns because blinding was not guaranteed. No study was classified as high risk for this domain. The reference standard was deemed low risk in all 40 studies, as the models were compared against established public datasets, ensuring a reliable and objective benchmark.

With respect to flow and timing, most studies were rated as low risk. However, one study was classified as high risk [4] due to lack of clarity on whether all patients underwent the same imaging protocols and whether assessments were blinded to clinical outcomes. Additionally, two studies [36, 54] were judged as having some concerns because insufficient details were provided on patient follow‐up and data handling procedures. Furthermore, no external validation was performed in these studies, as datasets were divided into training, validation and holdout test sets without further validation, which limits generalizability to broader clinical settings.

Applicability concerns were low in most studies. However, four studies were categorized as moderate risk [19, 36, 46, 54] due to variations in imaging protocols or patient demographics that may not fully align with the population being evaluated. Three studies were considered high risk [4, 7, 59], since the patient cohort differed substantially from the intended clinical application, reducing the relevance of its findings. While most studies had low applicability concerns, some demonstrated moderate to high risks due to differences in patient populations. The QUADAS‐2 assessment summary is presented in Supporting Information S1: Figure 3.

### Performance of AI in ACL tears diagnosis

We performed a pooled analysis of the 31 included studies to assess the overall performance of AI in the diagnosis of ACL tear. The pooled sensitivity (Figure 2a) and specificity (Figure 2b) were 94% (95% CI: 0.93–0.95, *I* ^2^ = 84.6%) and 93% (95% CI: 0.92–0.93, *I* ^2^ = 92.0%), respectively, both of which showed significant heterogeneity. The pooled DOR was 252.19 (95% CI: 137.44–462.74, *I* ^2^ = 90.9%). The DOR is significantly greater than 1, which indicates that AI has a good discrimination of ACL tears.

**Figure 2:**
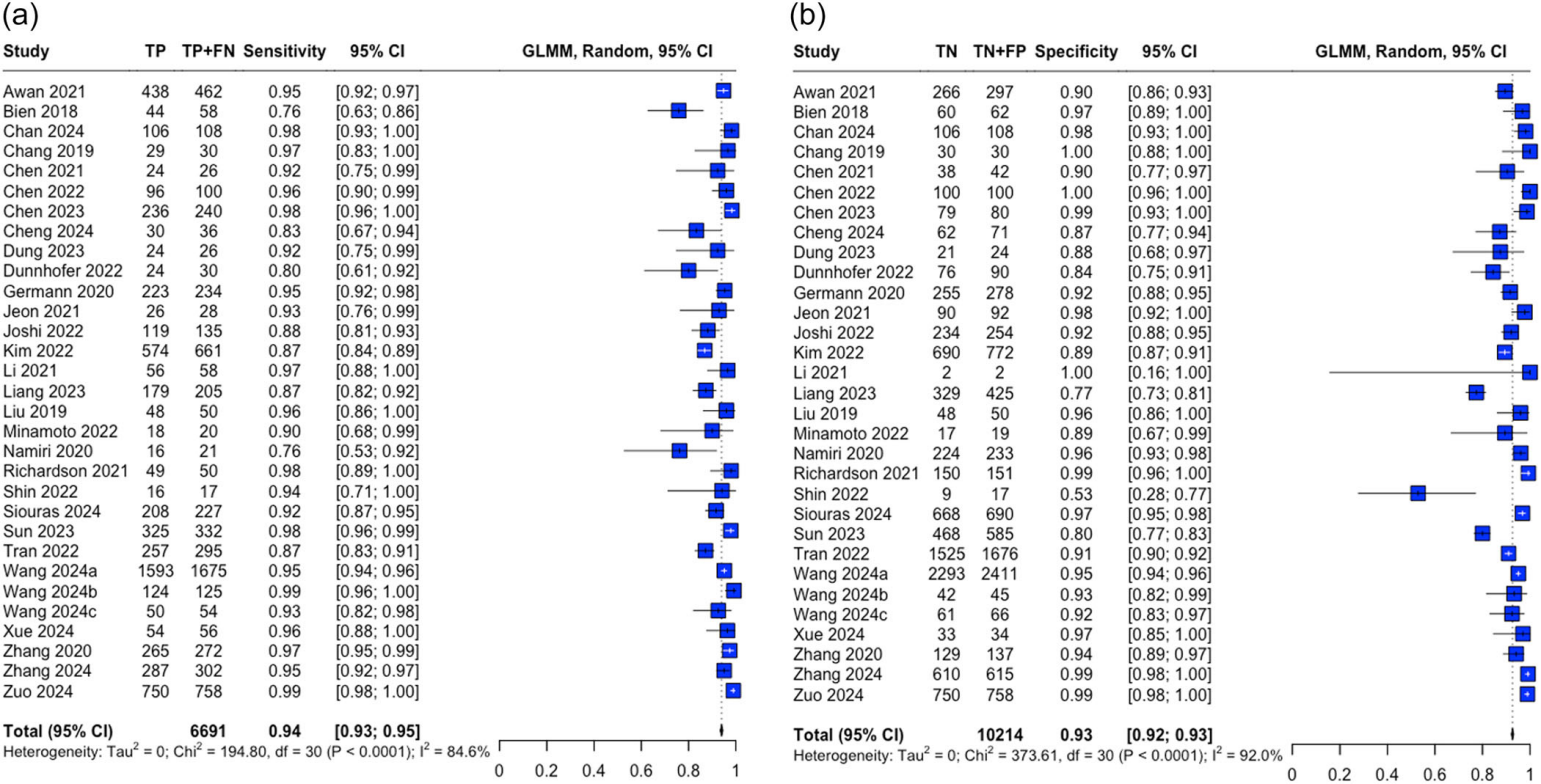
(a) Forest plot of sensitivity for AI in ACL tear diagnosis. (b) Forest plot of specificity for AI in ACL tear diagnosis. ACL, anterior cruciate ligament; AI, artificial intelligence; CI, confidence interval; FN, false negatives; FP, false positives; GLMM, generalized linear mixed model; TP, true positives.

The sROC curve is shown in Figure 3, and the AUC was 0.972 (95% CI: 0.949–0.974). This shows that AI has excellent performance in the diagnosis of ACL tears.

**Figure 3:**
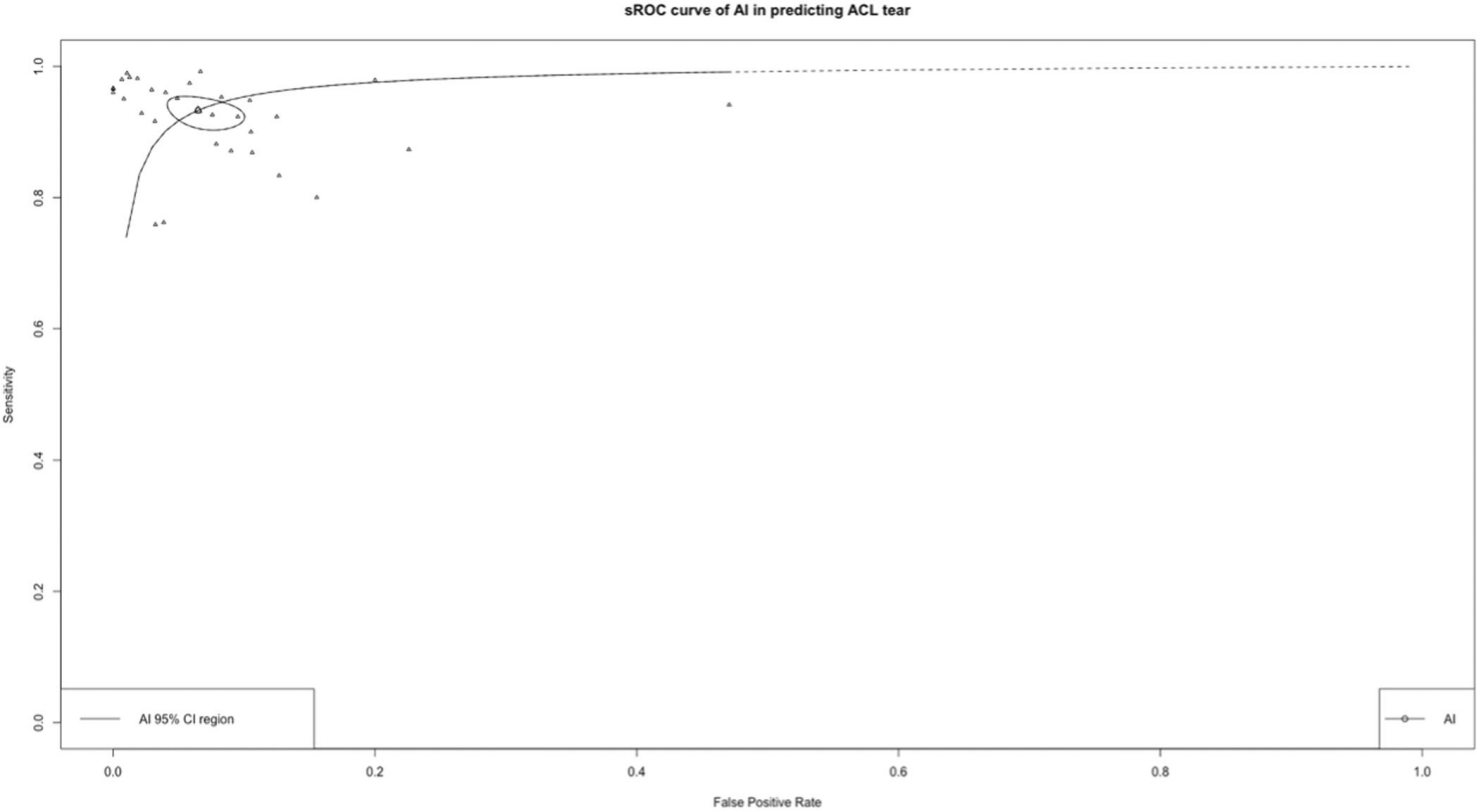
sROC curve of AI in predicting ACL tear. ACL, anterior cruciate ligament; AI, artificial intelligence; sROC, summary receiver operating characteristic.

### Publication bias

We used Deeks's funnel plot to evaluate publication bias [14]. As shown in Figure 4, there was no significant publication bias in the 31 studies included (*p* = 0.86).

**Figure 4:**
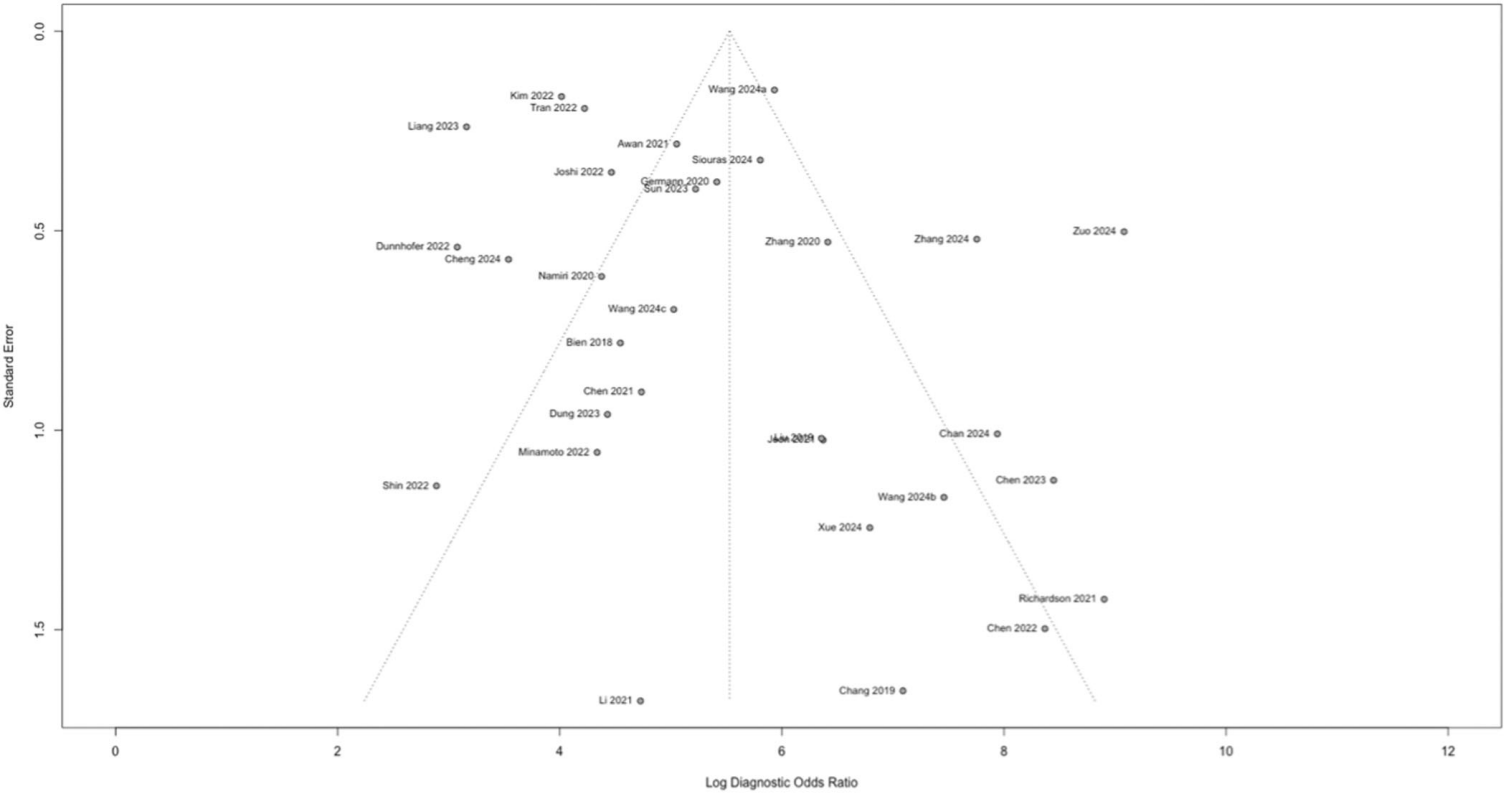
Funnel plot of sensitivity analyses.

### AI versus radiologists

Of the 31 studies included in the meta‐analysis, four compared AI with radiologists' performance [4, 9, 19, 29]. An essential condition for inclusion was that the same dataset had to be used for AI versus radiologists comparisons.

We pooled the data of the AI and Radiologists and performed a subgroup analysis. The results were as follows: sensitivity (Figure 5a) and specificity (Figure 5b) of AI in diagnosing ACL tears were respectively 0.90 (95% CI: 0.88–0.92) and 0.91 (95% CI: 0.89–0.92), reflecting a high level of diagnostic accuracy. High heterogeneity for sensitivity (*I* ^2^ = 91.8%) and moderate heterogeneity for specificity (*I* ^2^ = 64.9%) were observed, indicating variability in AI performance across different study settings and systems. Radiologists demonstrated comparable accuracy, achieving pooled sensitivity (Figure 6a) and specificity (Figure 6b) of 0.85 (95% CI: 0.83–0.87) and 0.90 (95% CI: 0.88–0.91), respectively. High heterogeneity (*I* ^2^ = 92.8% for sensitivity) and moderate heterogeneity (44.3% for specificity) among these studies also highlight some variability in the performance of radiologists.

**Figure 5:**
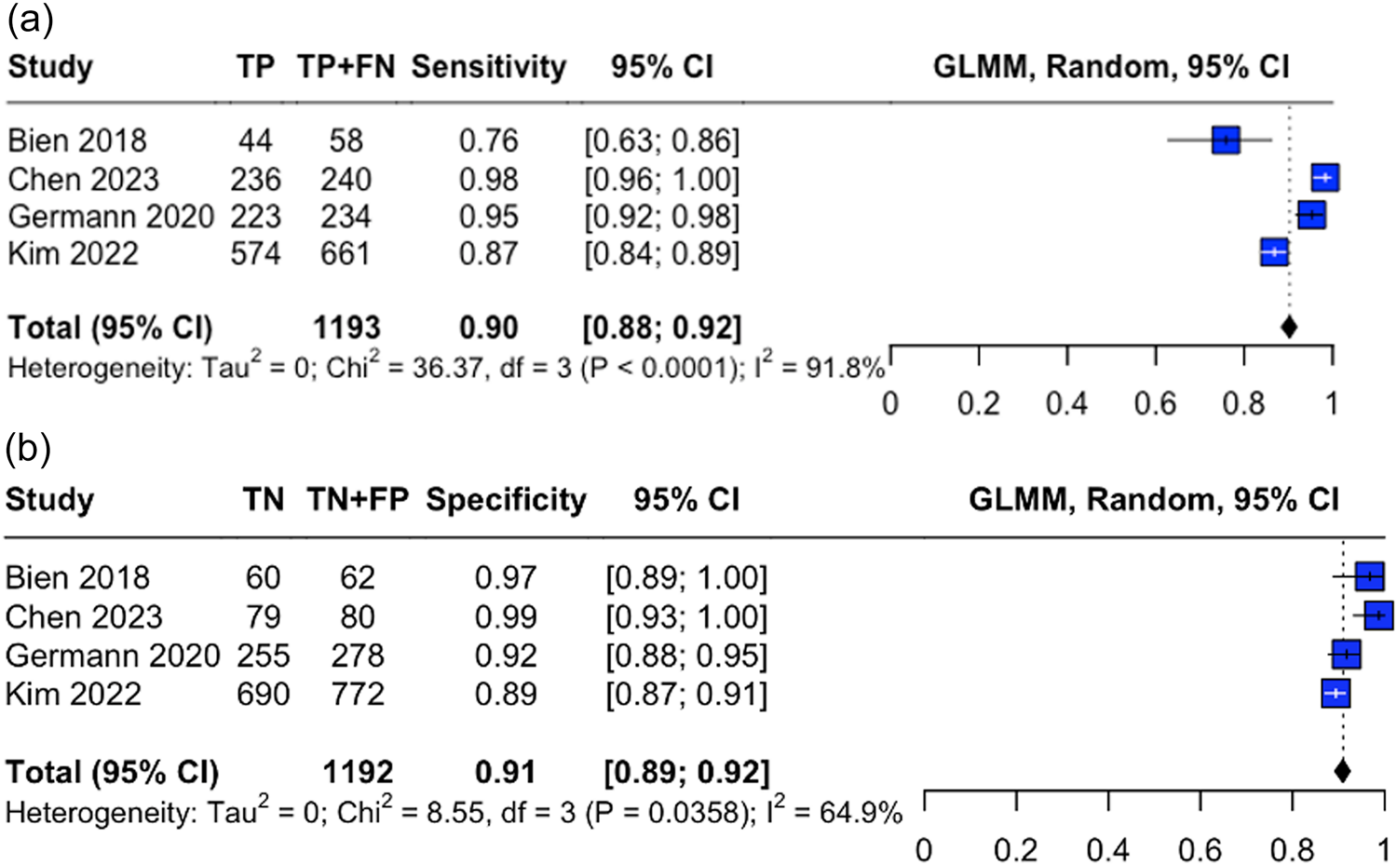
(a) AI sensitivity. (b) AI specificity. AI, artificial intelligence; CI, confidence interval; FN, false negatives; FP, false positives; GLMM, generalized linear mixed model; TP, true positives.

**Figure 6:**
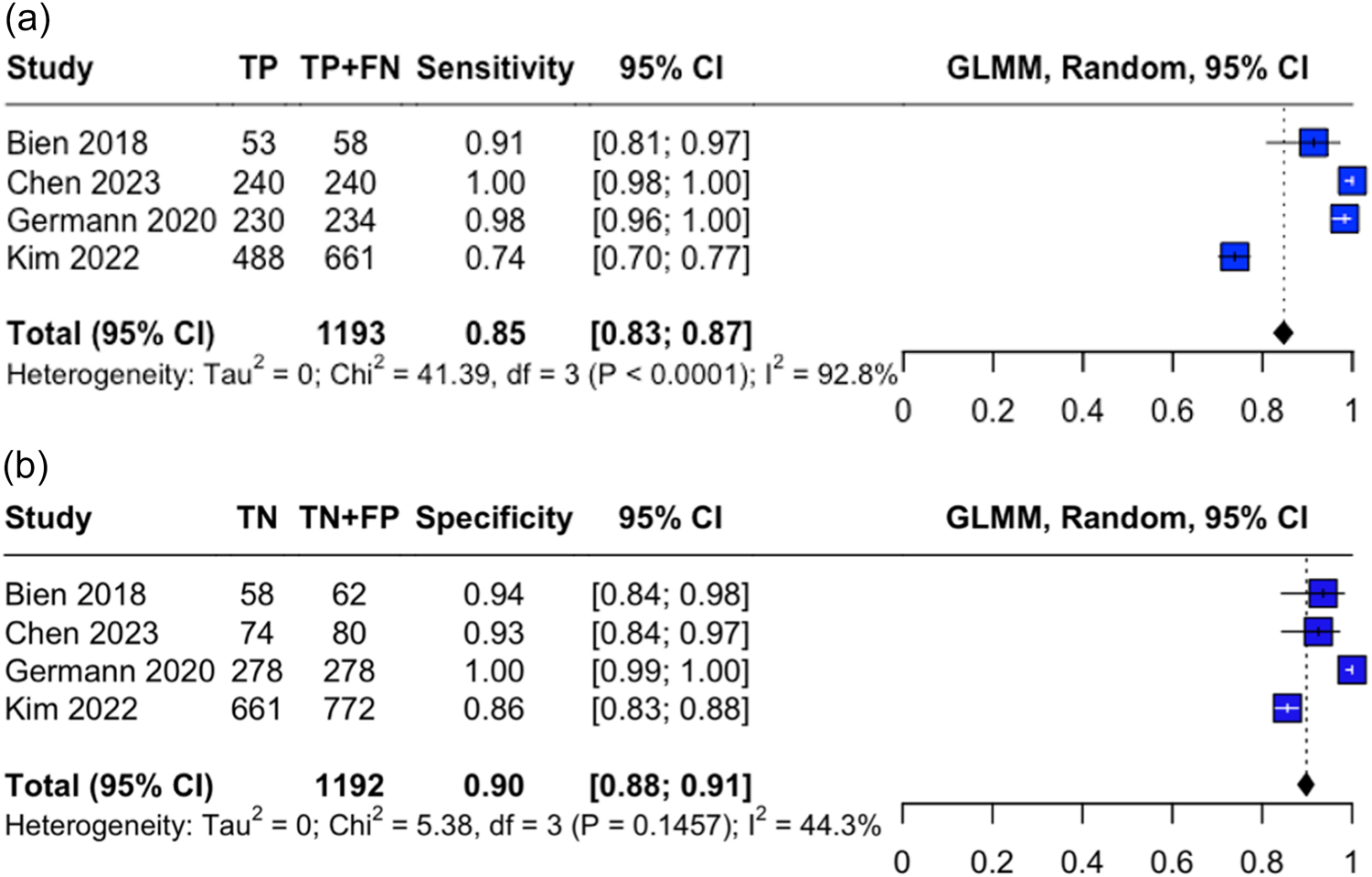
(a) Radiologist sensitivity. (b) Radiologist specificity. CI, confidence interval; FN, false negatives; FP, false positives; GLMM, generalized linear mixed model; TP, true positives.

The comparative analysis using the sROC curve revealed that both AI and radiologists achieved high diagnostic accuracy for ACL tears, with AI showing a slight edge (*p* = 0.782) (Figure 7). This analysis underscores AI's potential to match diagnostic precision in clinical settings, with no significant difference from traditional diagnostic methods employed by radiologists. AUC and DOR are comprehensive indicators of diagnostic performance, and larger values indicate stronger diagnostic capability of AI.

**Figure 7:**
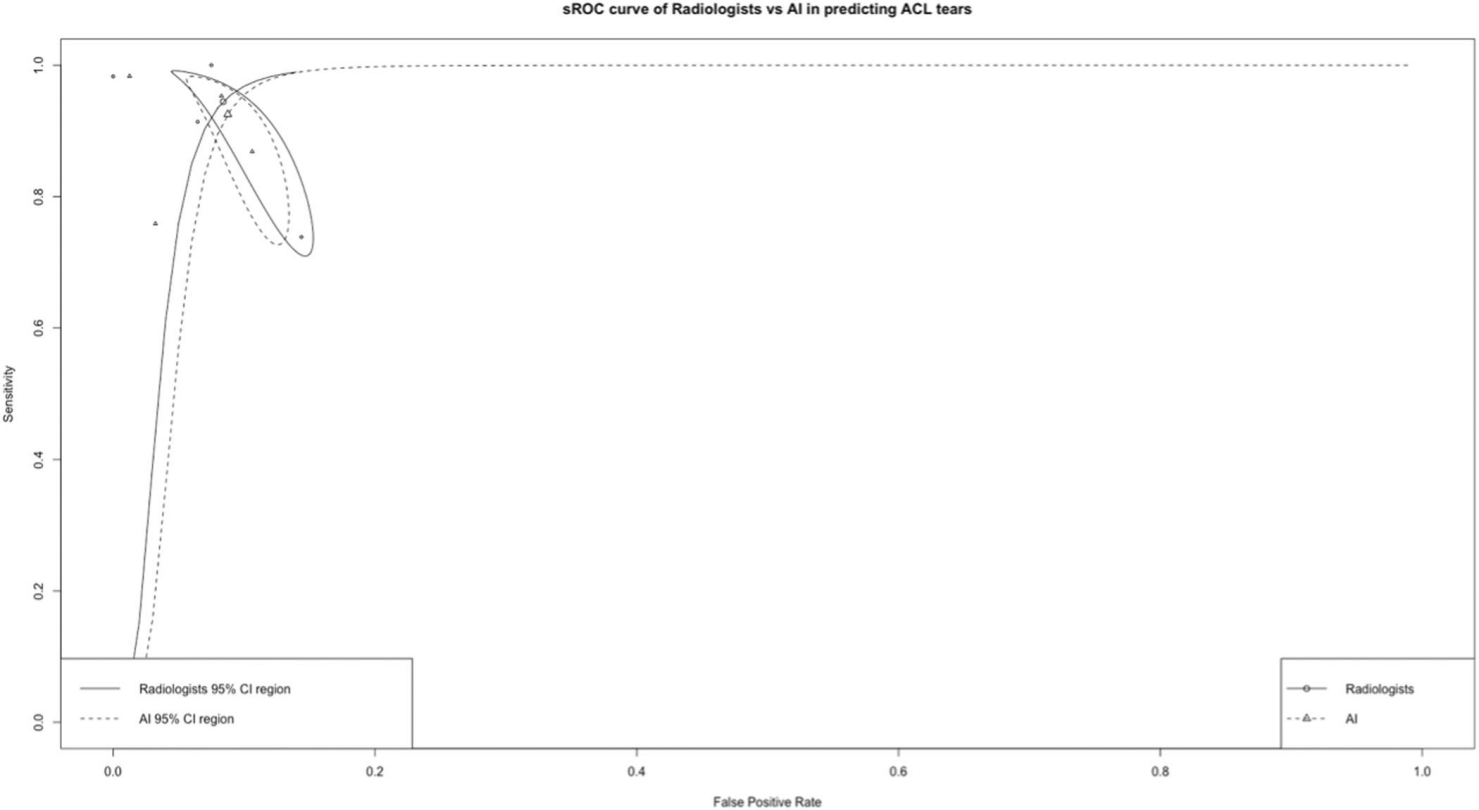
sROC curve of radiologists versus AI in predicting ACL tear. ACL, anterior cruciate ligament; AI, artificial intelligence; CI, confidence interval; sROC, summary receiver operating characteristic.

## DISCUSSION

The principal finding of this systematic review and meta‐analysis is that AI demonstrates excellent diagnostic accuracy for ACL tears, with an overall pooled sensitivity of 0.94 and specificity of 0.93. Furthermore, when directly compared to radiologists, AI models achieved statistically comparable performance (*p* = 0.782), highlighting their robust potential as reliable diagnostic adjuncts.

Our results closely align with the recent comprehensive meta‐analysis by Gill et al., which evaluated 52 AI models for ACL tear detection [21]. Gill et al. reported a pooled AI sensitivity of 0.907 and specificity of 0.913, which strongly mirrors our overall pooled findings. Moreover, their subgroup analysis comparing AI directly to clinicians found no statistically significant differences in diagnostic accuracy, completely corroborating our sROC findings. Both our study and Gill et al. observed high heterogeneity across the included literature (in our overall analysis, *I* ^2^ = 84.6% for sensitivity and 92.0% for specificity; in Gill et al., *I* ^2^ = 100% and >99.9%, respectively), underscoring that while AI performance is highly accurate, it remains sensitive to variations in dataset characteristics, reference standards and imaging protocols. Consequently, both analyses independently arrive at the same clinical consensus: AI is currently best positioned as a supportive tool to enhance clinician workflows rather than a standalone replacement.

Our findings are consistent with the growing body of literature supporting the role of AI in musculoskeletal imaging. For example, Zhao et al. analysed over 13,000 patients and more than 57,000 MRI images in a large review of AI applied to meniscal injuries [64]. They reported that algorithms performed slightly better in detecting meniscus tears (sensitivity 87%, specificity 89%) than in locating them (sensitivity 88%, specificity 84%). While prediction models showed promising diagnostic ability, their limitations in accurately identifying the tear's location highlight that AI performance is not uniform across tasks. This nuance underscores the importance of disease‐ and context‐specific evaluations, such as our dedicated analysis of ACL tears.

The strength of AI in ACL diagnosis is further supported by the scoping review of Fritz and Fritz, which concluded that DL algorithms often approach the accuracy of human readers in musculoskeletal MRI [17]. Importantly, Fritz noted that musculoskeletal radiologists still outperformed most DL algorithms in studies including a direct comparison. This is in line with our findings: although AI demonstrates excellent accuracy, we interpret that it should be seen as a complementary tool rather than a replacement. Another key difference is that Fritz's review covered a broad range of joint lesions, whereas our work provides focused evidence specifically for ACL tears, filling a gap that had not yet been addressed quantitatively.

Comparable results were also demonstrated by Bien et al., who applied a CNN to ACL diagnosis [4]. Their model achieved an AUC of 0.937 and specificity of 0.968, which closely mirror our pooled estimates. However, their reported sensitivity (0.759) was somewhat lower than the 0.939 observed in our analysis. Such variation may be explained by differences in datasets, image quality or algorithm design, but together both studies reinforce the potential of DL to support clinical decision‐making. These findings also validate the early predictions of Lao et al., who anticipated a central role for AI in ACL diagnostics long before large‐scale evidence became available [31].

Further reinforcing this potential, Herman et al. stressed the importance of explainable AI (XAI) to ensure clinical transparency and usability, an aspect increasingly recognized as critical for real‐world implementation [23]. Similarly, Garwood et al. and Bousson et al. emphasized AI's utility in automating musculoskeletal imaging tasks and improving accuracy in diagnosing conditions like fractures and ligament injuries [5, 18]. Santomartino et al. also called for academic radiology departments to lead AI adoption, underscoring the need for clinician involvement in development to ensure clinical relevance and trust [43].

In the specific context of ACL tear diagnosis, AI exhibited a level of precision statistically comparable to that of radiologists (*p* = 0.782, Figure 7). Despite this comparable performance, the varying levels of heterogeneity observed across studies (ranging from moderate to high) suggest that AI's diagnostic accuracy can vary depending on the system and clinical context. Therefore, while AI can achieve high levels of diagnostic accuracy (as illustrated by the sROC curve), it does not consistently surpass traditional radiological methods.

The potential clinical applications of AI are substantial, particularly in the context of primary care physicians (PCPs), who often serve as the first point of contact for patients with suspected ACL injuries. In regions with limited access to orthopaedic specialists, PCPs face the critical task of deciding whether to refer patients for further evaluation. AI‐based tools can assist this decision‐making process by providing accurate and timely diagnostic support, helping clinicians identify patients who truly require specialist referral. By functioning as a reliable triage mechanism, AI may reduce diagnostic delays, optimize referral pathways and alleviate the workload on orthopaedic and radiology services. Ultimately, the integration of AI into primary care workflows could enhance the overall efficiency and equity of musculoskeletal care delivery, particularly in underserved or resource‐constrained settings.

Beyond clinical practice, our findings have important implications for research and policy. The growing influence of AI in ACL tear diagnosis was recently emphasized by Gill et al., who analysed the 50 most cited studies from the past 7 years [22]. Their bibliometric analysis confirmed the robustness of current evidence, including our meta‐analytic results, while highlighting persistent barriers to clinical adoption. These include algorithmic bias, data privacy, explainability, cost‐effectiveness and interoperability. Furthermore, the underutilization of radiomic‐based models, despite their strong diagnostic potential, represents a critical research gap.

Therefore, future investigations should focus on developing XAI frameworks, conducting external multicenter validation studies and implementing standardized reporting and benchmarking guidelines. Policymakers and professional bodies will also need to establish regulatory standards and reimbursement models that facilitate safe, transparent and equitable AI integration into clinical workflows. Together, these efforts will be essential for translating AI's diagnostic accuracy into meaningful patient outcomes and sustainable healthcare innovation.

This study represents the first meta‐analysis to directly compare the diagnostic performance of AI models and radiologists in detecting ACL tears. Its strengths include a comprehensive search strategy across major databases, adherence to PRISMA‐DTA reporting standards and a rigorous dual‐reviewer screening process, all of which enhance methodological robustness and reliability.

However, several limitations should be acknowledged. First, this analysis focused exclusively on the diagnosis of ACL tears and did not assess lesion localization or severity grading, elements essential for surgical planning and prognosis. While the diagnostic accuracy of AI is encouraging, future models must address these dimensions to enhance their clinical applicability. Second, substantial heterogeneity was observed across studies, likely stemming from variations in dataset size, composition and source. Training datasets ranged from fewer than 200 MRI images from single centres to over 10,000 images from multicenter repositories. Additionally, a significant number of the included studies relied on the same public datasets, such as MRNet. This heavy reliance raises critical concerns regarding the presence of duplicate patient data across different studies, which may have impacted our pooled estimates. Furthermore, it introduces a substantial risk of data leakage, potentially leading to artificially inflated and overly optimistic performance estimates for AI models.

Finally, as highlighted by Corban et al., the ‘black box’ nature of AI remains a critical barrier to clinical adoption [13]. The lack of transparency and interpretability limits clinician trust and impedes real‐world implementation. Future research should prioritize the development of XAI frameworks to improve transparency, foster clinician confidence and facilitate safe integration into clinical workflows.

## CONCLUSION

This meta‐analysis demonstrates that AI models can accurately detect ACL tears on MRI with sensitivity and specificity comparable to radiologists, offering potential to support primary care physicians in decision‐making regarding specialist referrals. Future research should prioritize XAI frameworks, external validation and clinical workflow integration to address current barriers and ensure successful implementation in routine practice.

## AUTHOR CONTRIBUTIONS

Gabriel Moraes de Oliveira was responsible for study conceptualization, methodology design and overall supervision. Literature search and database retrieval were conducted by Gabriel Moraes de Oliveira, Serafina Zotter, Brian Tao and Ana Beatriz Nardelli da Silva. Title and abstract screening, full‐text review and data extraction were performed by Gabriel Moraes de Oliveira, Serafina Zotter, Brian Tao, Ana Beatriz Nardelli da Silva, Maria Mascarenhas, Chilan B. G. Leite and Guilherme Almeida. Risk of bias assessments were independently conducted by Gabriel Moraes de Oliveira, Ana Beatriz Nardelli da Silva and Giovanna Medina, with discrepancies resolved by consensus with Nacime Mansur. Data synthesis and statistical analysis were completed by Gabriel Moraes de Oliveira and Ana Beatriz Nardelli da Silva, with methodological oversight by Giovanna Medina. The initial draft of the manuscript was prepared by Gabriel Moraes de Oliveira and Ana Beatriz Nardelli da Silva, with critical revisions and intellectual input from all co‐authors. Technical and editorial feedback were provided by Nacime Mansur and Giovanna Medina, who also contributed to final manuscript approval. Project administration and coordination across institutions was managed by Gabriel Moraes de Oliveira. Gabriel Moraes de Oliveira is the guarantor and accepts full responsibility for the integrity of the work, the accuracy of the data and the decision to submit the manuscript for publication.

## CONFLICT OF INTEREST STATEMENT

The authors declare no conflicts of interest.

## ETHICS STATEMENT

The authors have nothing to report.

## Supporting information

RISMA‐DTA Checklist.

Supplementary Materials Figure 1. Complete search strategy for each database. Supplementary Materials Figure 2. Characteristics of the studies included comparing artificial intelligence (AI) and radiologists for anterior cruciate ligament (ACL) tear diagnosis. Supplementary Materials Figure 3. Estimation of Publication Bias. QUADAS‐2 assessment.

## Data Availability

All data supporting the findings of this systematic review (including extracted data and analyses) are available within the article (tables and figures) and in its Supporting Information. The included studies are publicly available in the literature. Additional data may be made available upon reasonable request to the corresponding author.
