# Supplementary material for: Artificial intelligence demonstrates comparable diagnostic accuracy to radiologists for anterior cruciate ligament tears on MRI: A systematic review and meta‐analysis
Source: Knee Surg Sports Traumatol Arthrosc. 2026 Jun 30;34(10):3816–28. doi: 10.1002/ksa.70478 (PMC13622925; doi:10.1002/ksa.70478)
Supplement: Supplementary file 2 [file KSA-34-3816-s002.docx]

**SUPPLEMENTARY MATERIALS FOR THE MANUSCRIPT**

***Artificial Intelligence vs. Radiologist on the Diagnosis for Anterior Cruciate Ligament Injury: A Systematic Review and Meta-Analysis***

**Supplementary Materials Figure 1**. Complete search strategy for each database.

**Supplementary Materials Figure 2.** Characteristics of the studies included comparing artificial intelligence (AI) and radiologists for anterior cruciate ligament (ACL) tear diagnosis.

**Supplementary Materials Figure 3**. Estimation of Publication Bias. QUADAS-2 assessment.

**Supplementary Materials Figure 1**. Complete search strategy for each database.

| **Database** | **Search Strategy** |
| --- | --- |
| **Cochrane** | (artificial intelligence OR machine learning OR deep learning OR "neural network") AND (ACL OR "anterior cruciate ligament") AND (MRI OR "magnetic resonance imaging") AND (“randomized controlled trial”:ti,ab,kw OR “controlled clinical trial”:ti,ab,kw OR randomized:ti,ab,kw OR placebo:ti,ab,kw OR “drug therapy”:ti,ab,kw OR randomly:ti,ab,kw OR trial:ti,ab,kw OR groups:ti,ab,kw) |
| **Embase** | ('artificial intelligence'/ti,ab OR 'machine learning'/ti,ab OR 'deep learning'/ti,ab OR 'neural network') AND (ACL OR 'anterior cruciate ligament') AND (MRI OR 'magnetic resonance imaging') AND (‘randomized controlled trial’/exp OR ‘controlled clinical trial’/exp OR randomized:ti,ab OR placebo:ti,ab OR randomly:ti,ab OR trial:ti,ab OR groups:ti,ab OR ‘randomized controlled trial’:ti,ab OR ‘controlled clinical trial’:ti,ab) |
| **PubMed** | (“artificial intelligence” OR “machine learning” OR “deep learning” OR “neural network”) AND (ACL or “anterior cruciate ligament”) AND (MRI OR “magnetic resonance”) AND ("randomized controlled trial"[pt] OR "controlled clinical trial"[pt] OR randomized[tiab] OR placebo[tiab] OR "drug therapy"[sh] OR randomly[tiab] OR trial[tiab] OR groups[tiab]) |

**Supplementary Materials Figure 2.** Characteristics of the studies comparing artificial intelligence (AI) and radiologists for anterior cruciate ligament (ACL) tear diagnosis.

| **Study (Year)** | **Country** | **Dataset Source** | **Data Acquisition Period** | **Study Design** | **Number of Patients** | **Mean Age (years, SD)** | **Number of MRI Exams** | **Algorithm** | **MRI Sequence(s)** | **Sensitivity** | **Specificity** | **Evaluation Metrics** |
| --- | --- | --- | --- | --- | --- | --- | --- | --- | --- | --- | --- | --- |
| Astuto, 2021 | USA | Univ. of California, Hospital for Special Surgery | 2011–2014 | Retrospective | 294 | 43 (15) | 1,435 | 3D CNN | 3D fast-spin-echo CUBE | 70–88% | 85–89% | AUC: 0.83–0.93; κ: improved intergrader agreement |
| Awan, 2021 | Malaysia | Clinical Hospital Centre Rijeka | 2007–2010 | Retrospective | 917 | NA | 917 | ResNet-14 CNN | Sagittal fat-suppressed PD | 91.6% | 94.6% | Accuracy: 92%; F1: 92.3% |
| Bien, 2018 | USA | Stanford Univ. Medical Center (MRNet) | 2001–2012 | Retrospective | 1,250 | 38 | 1,37 | CNN | Sag T2, Cor T1, Ax PD | NA | NA | AUC: 96.5% |
| Chan, 2024 | China | Stanford Univ. Medical Center (MRNet) | 2001–2012 | Retrospective | 1,250 | 38 | 1,37 | CNN | Coronal PDWI, T2WI | 98.49% | 98.30% | Accuracy: 97.9%; F1: 98.7% |
| Chang, 2019 | USA | Tertiary Medical Center (GE & Siemens) | 2013–2016 | Retrospective | 260 | 18–40 | 260 | CNN (dynamic patch sampling) | Coronal PD non-fat-suppressed FSE | 96.7% | 100% | Accuracy: >96% |
| Chen, 2021 | China | Hospital of Tianjin Univ. | 2020–2022 | Prospective cohort | 68 | 46.1 (15.3) | 68 | LASSO, RF, SVM | T1WI | 57% (SVM) | 79% (SVM) | Accuracy: 71% |
| Chen, 2022 | China | Taipei Veterans General Hospital | 2013–2017 | Retrospective | 800 | NA | 2,223 | CNN | Sagittal | 93.44% | 97.43% | Accuracy: 99.5%; F1: 89.8% |
| Chen, 2023 | China | First Affiliated Hospital of Soochow Univ. | 2019–2021 | Retrospective | 235 | 37.9 (14.8) | 665 | Transfer learning (EfficientNet-B0) | Thin-slice oblique sagittal FS-PDWI | 90–100% | 97–100% | κ (AI=0.946 vs Rad=0.838); Accuracy (AI=0.959 vs Rad=0.878) |
| Cheng, 2024 | China | First Affiliated Hospital of Wannan Med. College | 2019–2023 | Retrospective | 526 | 43.4 (14.6) | 2,032 | LASSO, SVM | T1WI, PDWI-FS | 93.3% (train), 85.7% (val) | 93% (train), 82.9% (val) | AUC: 0.973 (train), 0.927 (val) |
| Dung, 2023 | Vietnam | Hospital 1, Da Nang | 2018–2021 | Retrospective | 297 | 37.5 (1.4) | 303 | DCLU-NET | Sagittal fat-suppressed PD | NA | NA | Accuracy: 82–92% |
| Dunnhofer, 2022 | USA | Stanford Univ. Medical Center (MRNet) | 2001–2012 | Retrospective | 1,250 | 38 | 1,37 | CNN / MRPyrNET | Coronal PDWI, T2WI | 69–81.5% | 93.9% | AUC: 0.943–0.972 |
| Germann, 2020 | Germany | 59 institutions → single center | 2015–2019 | Retrospective | 512 | 34 (15) | 5,802 | D-CNN | Sag & Cor fat-suppressed | 96.1% | 93.1% | AUC: 0.935 |
| Kim, 2022 | Korea | Boramae & Konkuk Univ. Med. Centers | 2010–2020 | Retrospective | 1,433 | 27.3 (8.7) | 1,433 | CNN | NA | 86.8% | 89.4% | AUC: 0.927 |
| Li, 2021 | China | Peking Univ. Shenzhen Hospital | 2019–2020 | Cross-sectional | 30 | 37.8 (5.2) | NA | CNN | Sag T2WI SPAIR | 96.8% | 90.6% | Accuracy: 92.2% |
| Li, 2023 | China | Ningbo General Hosp. + MRNet | 2001–2021 | Retrospective | 1,35 | NA | 1,47 | 3D Weighted Multi-View CNN | T1W, T2W, PD-FatSat (all planes) | NA | NA | AUC: 97% (MRI-ACL), 92.9% (MRNet) |
| Liang, 2023 | China | Harbin Med. Univ. | 2012–2020 | Retrospective | 313 | 38.5 (12.1) | 630 | 13-layer ResNet CNN (attention) | PD-FS, T2-FS, T1W, STIR | 92.7% | 65.1% | AUC: 0.8886 |
| Liu, 2019 | USA | Internal dataset | 2010–2017 | Retrospective | 350 | 27.5 / 39.4 | 350 | Dual CNNs | Sagittal PD + T2-FS | 96% | 96% | AUC: 0.98 |
| Mei, 2022 | USA | RadImageNet | 2005–2020 | Retrospective | 1,021 | NA | 179,555 | Inception-ResNet-v2, etc. | NA | ~95% | ~95% | AUC: 0.91–0.97 |
| Minamoto, 2022 | Japan | PACS | 2009–2019 | Retrospective | 193 | 27.2 / 26.1 | 200 | CNN | Sagittal T1W-FS | 91% | 86% | Accuracy: 88.5% |
| Namiri, 2020 | USA | GE/NIH | 2011–2014 | Retrospective | 224 | 47 (14) | 1,243 | 2D & 3D CNN | 3D PD-FS | 76–93% | 88–100% | Accuracy: 89–92% |
| Jeon, 2021 | Singapore | Chiba + Stanford | 2014–2018 2001–2012 | Retrospective | 1,250 | 38 | 2,547 | Lightweight 3D CNN (attention) | NA | 0.95 | 0.94 | AUC: 0.963–0.977 |
| Joshi, 2022a | India | MRNet dataset | NA | Retrospective | NA | NA | 1,25 | CPDCNN | Sag T1, T2, PD-FS | 96.7% | 96.5% | Accuracy: 96.6%; F1: 0.96 |
| Joshi, 2022b | India | MRNet dataset | NA | Retrospective | NA | NA | 1,295 | MNLBP + KNN/SVM | Sag T2, Cor T1, Ax PD | 87–88% | 92% | Accuracy: 89% |
| Qiu, 2024 | China | 5 Chinese clinical centers | NA | Retrospective | 1,748 | NA | 8,74 | CoPAS CNN | PDW (multi-plane), T1W, T2W | NA | NA | AUC: 0.812; Accuracy: 0.65–0.80 |
| Richardson, 2021 | USA | Institutional PACS | 2016–2019 | Retrospective | 2,007 | 33–44 | 4,014 | CNN | Sagittal FS/NFS | 94.4% | 94.3% | NA |
| Shin, 2022 | Korea | Yeungnam Univ. Hospital | 2010–2020 | Retrospective | 164 | 43.6 (17.5) | 164 | VGGNet CNN | Oblique-sagittal T2-FS | NA | NA | AUC: 94.1% |
| Siouras, 2024 | Greece | MRNet, KneeMRI, UGHL | 2001–present | Mixed | 1,581 | NA | 1,631 | Hybrid deep learning | PDW-FS, T1W, T2W | 91.7–96.3% | 92–96.8% | Accuracy: 83–89% |
| Sridhar, 2022 | India | MRNet (Stanford) | 2001–2012 | Retrospective | 535 | NA | 1,37 | Inception-v3 DTL CNN | Coronal PDWI, T2WI | NA | NA | Accuracy: 95–99% |
| Stajduhar, 2017 | Croatia | Clinical Hospital Centre Rijeka | 2007–2014 | Retrospective | 917 | NA | 917 | SVM, Random Forest | Sag, Cor, Ax PDW-FS | NA | NA | AUC: 0.894–0.943 |
| Sun, 2023 | Malaysia | Clinical Hospital Centre Rijeka | 2007–2010 | Retrospective | NA | NA | 917 | DBN, ResNet-14 CNN | Sag PDW-FS | 98% | 80% | Accuracy: 96% |
| Tran, 2022 | France | MRNet, KneeMRI | 2017–2018 | Retrospective | 17,738 | NA | 19,765 | NLP | Coronal & Sag PD/T2W-FS | 68–87.5% | 89–93% | AUC: 0.922–0.962 |
| Wang M., 2024 | China | OAI + Internal dataset | 10-year follow-up | Retrospective | 6,552 | 49–75 | 11,181 | ResNet-18 CNN | Sagittal IW-FSE | 83–94% | 93–99% | AUC: 0.94–0.99 |
| Wang X., 2024 | China | MRNet (Stanford) | 2001–2012 | Retrospective | 1,199 | 36–38 | 1,25 | ResNet SGNet | Multi-view PD/T2W | 92.6% | 92.4% | AUC: 0.9747 |
| Wang D., 2024 | China | MRNet (Stanford) | NA | Retrospective | NA | NA | 1,37 | InceptionV4 + FO-LOA | NA | 98% | 98% | F1: 98%; MCC: 88% |
| Wang J., 2024 | China | Third Xiangya Hospital | 2021–2022 | Retrospective | 147 | <57 | 1,652 | ACGNN + CRF | Oblique coronal, sagittal | NA | NA | Accuracy: 92.9% (cor), 91.9% (sag) |
| Wang DY, 2024 | China | 5 Chinese centers | 2021–2022 | Retrospective | NA | 39.4 (4–90) | 26,853 | ResNet CNN | Sag/Cor PDW, T2W | 95.1% | 95.1% | AUC: 0.987 |
| Zhang, 2020 | China | Third Affiliated Hosp. of Southern Med. Univ. | 2011–2019 | Retrospective | 409 | 40.9 (16) | 408 | 3D DenseNet | Sag PDW-SPAIR | 97.6% | 94.4% | Accuracy: 95.7% |
| Zhang, 2024 | China | Clinical Hospital Centre Rijeka | 2006–2014 | Retrospective | NA | NA | 917 | CNN + MGSA | PDW-FS | NA | NA | Accuracy: 99.6% |
| Zou, 2024 | China | Stanford Univ. Medical Center (MRNet) | 2001–2012 | Retrospective | 1,370 | NA | 1,37 | Optimized GRU + FFDO | Sag/Cor PDW-FS, Ax T2W-FS | NA | NA | Accuracy: 97.95% |
| Xue, 2024 | China | Hunan Provincial People’s Hospital | 2019–2022 | Retrospective | 862 | 36.8 (18–78) | 862 | ACL-DNet, ACL-SNet | Sag PDW-SPAIR | 97% | 97% | AUC (Experts): 88–96% |

**Abbreviations:** AI, artificial intelligence; ACL, anterior cruciate ligament; AUC, area under the curve; CNN, convolutional neural network; DNN, deep neural network; MRI, magnetic resonance imaging; PD, proton density; PDW, proton-density-weighted; PDW-FS, fat-suppressed proton-density-weighted; T1W/T2W, T1- or T2-weighted sequence; FSE, fast-spin-echo; FS, fat-suppressed; SPAIR, spectral attenuated inversion recovery; ROC, receiver operating characteristic; Sag, sagittal; Cor, coronal; Ax, axial; SVM, support vector machine; RF, random forest; SD, standard deviation; NA, not available

**Supplementary Materials Figure 3**. QUADAS-2 assessment.
